# High-Throughput Profiling of Anti-Glycan Humoral Responses to SIV Vaccination and Challenge

**DOI:** 10.1371/journal.pone.0075302

**Published:** 2013-09-23

**Authors:** Christopher T. Campbell, Sean R. Llewellyn, Thorsten Damberg, Ian L. Morgan, Marjorie Robert-Guroff, Jeffrey C. Gildersleeve

**Affiliations:** 1 Chemical Biology Laboratory, National Cancer Institute, National Institutes of Health, Frederick, Maryland, United States of America; 2 Vaccine Branch, National Cancer Institute, National Institutes of Health, Bethesda, Maryland, United States of America; University of Massachusetts Medical Center, United States of America

## Abstract

Recent progress toward an HIV vaccine highlights both the potential of vaccines to end the AIDS pandemic and the need to boost efficacy by incorporating additional vaccine strategies. Although many aspects of the immune response can contribute to vaccine efficacy, the key factors have not been defined fully yet. A particular area that may yield new insights is anti-glycan immune responses, such as those against the glycan shield that HIV uses to evade the immune system. In this study, we used glycan microarray technology to evaluate anti-glycan antibody responses induced by SIV vaccination and infection in a non-human primate model of HIV infection. This comprehensive profiling of circulating anti-glycan antibodies found changes in anti-glycan antibody levels after both vaccination with the Ad5hr-SIV vaccine and SIV infection. Notably, SIV infection produced generalized declines in anti-glycan IgM antibodies in a number of animals. Additionally, some infected animals generated antibodies to the Tn antigen, which is a cryptic tumor-associated antigen exposed by premature termination of *O*-linked glycans; however, the Ad5hr-SIV vaccine did not induce anti-Tn IgG antibodies. Overall, this study demonstrates the potential contributions that glycan microarrays can make for HIV vaccine development.

## Introduction

A vaccine for HIV offers the best prospects for ending the AIDS pandemic, and intense efforts have been directed toward HIV vaccine development. This work has culminated in a recent study that demonstrated the first evidence of efficacy in a phase III clinical trial for an HIV vaccine [1]. While this groundbreaking study has shown that an HIV vaccine can provide protection from infection, the overall efficacy was modest and significant improvements still are urgently needed.

Many aspects of the immune response can contribute to vaccine efficacy, but the key factors have not been defined fully yet. One area that has been largely understudied is immune response to glycans, which can play important roles in immune responses to HIV. For example, HIV has a “glycan shield”, which consists of self glycans that help to camouflage key HIV protein antigens [2], [3]. Over the last few years, many broadly HIV neutralizing antibodies have been discovered, and a number of these bind glycans or bind in a glycan-dependent manner such as 2G12 [4], PGT121 [5], and PG9/PG16 [6]. 2G12, for instance, binds to Manα1-2Man on complex high-mannose N-glycans, which help to shield critical epitopes on gp120 [7]. Similarly, high-mannose as well as complex-type N-glycans are recognized by the recently identified V3 loop binding antibody, PGT121 [8]. These findings suggest that serum anti-glycan antibodies may be critical for preventing and controlling HIV infection, and carbohydrate-targeted HIV vaccines are under development [9], [10]. Nevertheless, the extent of immune responses to glycans – both components of the glycan shield as well as other glycans – has not been characterized fully. Due to the technical challenges associated with analysis of complex mixtures of serum anti-glycan antibodies, there has been no comprehensive characterization of how HIV vaccines or infection alter levels of antibodies to diverse glycans in humans.

Glycan microarray technology [11], [12] provides a comprehensive overview of anti-glycan antibody repertoires while using only minimal amounts of valuable glycans and serum samples (2–4 µL). Glycan microarrays contain hundreds of different carbohydrates immobilized on a solid support and provide a high-throughput tool to profile anti-glycan antibody responses. This technology has been used extensively to evaluate binding properties of monoclonal antibodies [13],[14] and lectins [15], [16], [17] and to identify serum antibodies as potential biomarkers for cancer, infections, and autoimmune diseases (S. M. Muthana and J. C. Gildersleeve, in press). In addition, it has been used to profile immune responses to vaccines [18]. Glycan microarray technology has been used to profile immune responses to potential HIV vaccines in rabbits [19], [20], [21], but it has not yet been used to profile immune responses to SIV vaccines or SIV infection in a non-human primate model of HIV infection.

In this study, we used glycan microarray technology to evaluate anti-glycan antibody responses induced by an SIV vaccine and by SIV infection in the rhesus macaque non-human primate model of HIV infection [22], [23]. Simian immunodeficiency virus (SIV) infects CD4^+^ T-cells through interactions with CD4 and its main co-receptor CCR5. Like in humans, infection progresses to AIDS. In a previous study, a promising vaccine composed of mucosal priming with replication-competent Adenovirus type 5 host range mutant (Ad5hr) recombinants encoding SIV*env/rev*, and/or SIV*gag* and/or SIV*nef* followed by boosting with native SIV gp120 protein or a polypeptide representing the CD4 binding site of the viral envelope was shown to elicit strong humoral and cellular immune responses to the immunizing antigens. Protection against SIV_mac251_ intrarectal challenge was evident in 39% of the macaques by potently reduced plasma viremia [24]. Interestingly, several vaccinated animals exhibited “elite control” of viremia more than 6.5 years after initial immunization, even after an additional viral challenge, CD8^+^ T cell depletion and further heterologous viral challenge [24], [25]. As protection in these macaques was shown to be correlated with high titered anti-envelope antibody responses [24], [26], stored sera and plasma collected from these macaques provided an opportunity to explore the development of anti-glycan antibodies with vaccination and their possible role in vaccine induced protection. We profiled serum anti-glycan antibodies with a glycan microarray containing a diverse library of glycans in order to comprehensively characterize humoral responses to glycans. More in-depth studies are warranted to understand the functional role of the significant changes we found in antibody levels to diverse glycans in this non-human primate model.

## Materials and Methods

### Animals, vaccination regimen, and SIV challenge

The rhesus macaques (*Macaca mulatta*) used in this study were housed in accordance with the recommendations of the Association for Assessment and Accreditation of Laboratory Animal Care International Standards and with the recommendations in the Guide for the Care and Use of Laboratory Animals of the United States - National Institutes of Health. The Institutional Animal Use and Care Committee of BIOQUAL approved these experiments. When immobilization was necessary, the animals were injected intramuscularly with 10 mg/kg of ketamine HCl (Parke-Davis, Morris Plains N.J.). All efforts were made to minimize suffering. Details of animal welfare and steps taken to ameliorate suffering were in accordance with the recommendations of the Weatherall report, “The use of non-human primates in research”. Animals were housed in an air-conditioned facility with an ambient temperature of 21–25°C, a relative humidity of 40%–60% and a 12 h light/dark cycle. Animals were socially housed when possible or individually housed if no compatible pairing could be found. The animals were housed in suspended stainless steel wire-bottomed cages and provided with a commercial primate diet and fresh fruit twice daily, with water freely available at all times. All animals were monitored twice daily for activity, food and water intake, and overall health. Animals that reached IACUC defined endpoints, including pain or distress, that could not be alleviated therapeutically were humanely euthanized with an overdose of barbiturate consistent with the recommendation of the American Veterinary Medical Association.

**Figure 1:**
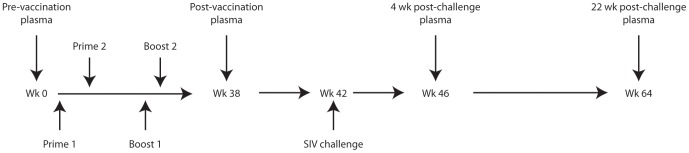
Immunization schedule for the Ad5hr-SIV trial. Each of the macaques underwent the same schedule. Ad5hr-SIV recombinant immunogens were administered at weeks 0 and 12 followed by envelope boosts at weeks 24 and 36. Intrarectal SIV_mac251_ challenge occurred at week 42. Blood serum and plasma samples were collected at weeks 0, 38, 46, and 64.

In a previously reported study [27], 48 juvenile rhesus macaques [*Macaca mulatta*; male (n = 28) and female (n = 20)] received replicating Ad5hr-SIV vaccine encoding one or more of three SIV genes: *env/rev*, *gag*, and *nef*. Boosters contained either native SIV_mac251_ gp120 in monophosphoryl lipid A-stable emulsion (MPL-SE) or an SIV_mac251_ peptomer (a repeating peptide polymer composed of 18-mer peptides representing the CD4 binding site of gp120) in phosphate buffered saline (PBS). Native SIV_mac251_ gp120, which was purified from cultured cells infected with SIV [28], [29], is glycosylated, although its glycans have not fully characterized. MPL-SE is a non-glycosylated adjuvant derived from lipid A [30]. As summarized in Table 1, each macaque received one of six possible combinations of Ad5hr-recombinants (administered at weeks 0 and 12), protein boosters (administered at weeks 24 and 36), and/or adjuvants. Vaccination required 36 weeks (Figure 1). 47 macaques completed the vaccination regimen, and one animal died prematurely of renal failure unrelated to the vaccine. At week 42 of the trial, macaques were rectally challenged with 10 50% monkey infectious doses (MID_50_) of pathogenic SIV_mac251_. All macaques tested negative for prior exposure to SIV, simian retrovirus type D, and simian T-cell leukemia virus. Animal maintenance and experimental procedures were conducted according to NIH guidelines.

**Table 1:** Vaccine and control groups for Ad5hr-SIV trial.

		Prime^1^				Boost^2^			
Group	No. of Macaques	Ad5hr-SIV env/rev	Ad5hr-SIV gag	Ad5hr-SIV nef	Ad5hr Vector	SIV gp120	SIV peptomer	MPL-SE	PBS
1	1	+				+		+	
2	6	+	+			+		+	
3	8	+		+		+		+	
4	8	+	+	+		+		+	
5	8	+					+		+
6	7				+			+	

1 Prime includes injections at week 0 (oral and intranasal) and week 12 (intratracheal).

2 Boost refers to injections at week 24 and 36 (both intramuscular).

Plasma and/or serum samples from 38 macaques (Table 1) in the Ad5hr-SIV vaccine trial were available for retrospective analysis. Blood samples were collected immediately prior to vaccination (week 0), 38 weeks post-vaccination (week 38), 4 weeks post-challenge (week 46), and 22 weeks post-challenge (week 64) (Figure 1). Reference serum pooled from 15 macaques was purchased from BioChemed Services (Winchester, VA). Prior to microarray analysis, all plasma and sera were stored at −80°C except during a brief heat inactivation (56°C for 30 minutes) to inactivate SIV virions [31]. (See Figure S1 in File S1 for verification that heat inactivation did not alter antibody binding.)

### Human serum samples

Sera were obtained from healthy, non-vaccinated humans confirmed to be HIV negative (n = 30; ProMedDx). All sera were frozen at −80°C prior to use.

### High-throughput profiling of plasma anti-glycan antibodies

Glycan microarrays consisted of 220 features printed in duplicate onto epoxide coated slides (SME2, Arrayit, Sunnyvale, CA) as previously described [32]. The array previously has been validated with several monoclonal antibodies and lectins [13], [14], [15], and it has been shown to have excellent reproducibility [33]. Briefly, the array contained neoglycoconjugates and glycoproteins. Neoglycoconjugates consist of a carrier protein (i.e., BSA or HSA) conjugated to multiple copies of a glycan. (Please see the supplemental glycan microarray data File S2 for details on the carrier protein, conjugation method, and density of glycans conjugated to the carrier.) Microarrays were printed in a 16 well format with all 220 components in duplicate using a Microgrid II robotic arrayer (Digilab Inc., Holliston, MA) and quilled pins (SMP2, Arrayit, Sunnyvale, CA). Printed arrays were vacuum sealed and stored at −20°C until required for assays.

Prior to use in assays, slides were checked for missing spots by pre-scanning for fluorescent DyLight 649 (Thermo Scientific) included in the print buffer (0.7 µg/mL). Slides were prepared for assays using slide modules (Grace Bio-Labs) that divided slides into 16 wells. Next, 200 µL 3% BSA in PBS (pH 7.4) was added to each well for overnight blocking at 4°C. After washing 6 times with PBS+0.05% Tween 20 (PBST, pH 7.4), 100 µL of diluted plasma or sera (1∶100 for macaques and 1∶50 for humans into 3% BSA+1% HSA in PBST, pH 7.4) were incubated on the array for 4 hours at 37°C and 100 RPM. One well per slide was dedicated to a reference serum sample (diluted 1∶50) that was analyzed alongside the plasma samples as a quality control and to aid in comparing data collected on different slides. All plasma samples were analyzed in duplicate wells on two slides in order to control for inter-slide variability. After incubation, arrays were washed 3 times with 200 µL PBST (pH 7.4). Arrays then were incubated with fluorescently tagged iso-type specific secondaries [100 µL 1% BSA+3% HSA+2 µg/mL TRITC labeled anti-monkey IgM or IgG (Brookwood Biomedical, Table S1 in File S1) in PBS, pH 7.4] for 2 hours at 37°C and 100 RPM. After washing 7 times in PBST (pH 7.4) and spin drying (1000 RPM for 5 minutes), arrays were scanned with a fluorescence scanner (GenePix 4000A, Molecular Devices).

### Processing of glycan microarray data

Fluorescence indicative of bound antibody was detected and quantified using GenePix Pro 6.0 software, which calculated the median pixel intensity for each array component corrected for the background signal. As described previously [32], pre-processing using Microsoft Excel determined the log_2_ value of the average of 4 spots collected from two slides. A reference serum sample was used to monitor inter-slide variability. Statistical analysis was performed with Partek Genomics Suite and Microsoft Excel. All glycan microarray data, including a full description of array components, will be made publically available at the Consortium for Functional Glycomics (CFG) website.

### Measurement of total immunoglobulin levels

Levels of total IgG, IgM, and IgA in plasma and serum samples were measured as follows. MagPlex carboxylated beads (Bio-Rad, CA) region 29, 37, 44 were conjugated to goat anti-IgG (gamma-chain specific), goat anti-IgA (alpha-chain specific) (both from Alpha Diagnostic International, TX) or goat anti-IgM (mu-chain specific) (Rockland, PA) antibodies respectively, following the coupling instructions of the Bio-Rad Amine coupling Kit. Anti-IgA, -IgG and -IgM beads were counted using disposable plastic cell counting chambers (Cellometer, Nexcelom Bioscience) and mixed in equal amounts. Standards for Rhesus IgG and IgA were obtained from the NIH Non-human Primate Reagent Resource program and Rhesus IgM standard was purchased from Alpha Diagnostics International. Approximately 2,000 beads/region/well were washed by aspirating the supernatant of the magnetically settled beads and washing them with 500 µL PBS containing 1% BSA (PBS/1% BSA). After aspiration of the wash buffer, beads were resuspended in 5 mL PBS/1% BSA. 50 µL of bead suspension were pipetted into wells of 96-well tissue culture plates, and 50 µL of standards (ranging from 1 µg/mL to 1 ng/mL) or serum/plasma dilutions were added in duplicate wells (final volume 100 µL). The plate was incubated for 30 min on a shaker in the dark at RT. Sample and standard wells were washed once with 100 µL of PBS/Tween20 (0.05%), and 100 µL detection antibody mix (biotinylated goat anti Rhesus IgG, IgA and IgM at a 1∶1000 dilution) in PBS/1% BSA plus Tween20 (0.05%) were added to each well and incubated as above. Wells were washed twice as described, and 50 µL of a 1∶500 dilution of Streptavidin-PE in PBS/BSA/Tween20 was added to each well and incubated for 10 min in the dark without agitation. Wells were washed once and beads were resuspended in 125 µL in PBS/BSA and a minimum of 50 beads per region were acquired on a BioPlex 200 system equipped with Bio-Plex Manager 4 software. Data were exported as an Excel file after background subtraction. Immunoglobulin levels were derived from standard curves generated in GraphPad Prism 5.01. Data from 2 experiments were averaged, and plots were generated in GraphPad.

### Viral loads and CD4^+^ T-cell levels

Viral loads and levels of peripheral CD4^+^ T cells were measured at overall week 64 as previously described [25].

## Results

### Similarly diverse anti-glycan antibody repertoires in healthy humans and macaques

Although SIV infected macaques are an established model for HIV infection of humans, we first compared the anti-glycan antibody profiles of humans and macaques to confirm that macaques are a relevant model for this aspect of humoral immunity. Our group [13], [33], [34], [35] and others [16], [17], [36], [37], [38], [39], [40], [41], [42], [43], [44], [45] have used glycan array technology to profile repertoires of serum antibodies in humans, but much less is known about macaque antibody repertoires and the optimal methods for measuring their levels on a glycan array. In addition, the macaque samples available for this study were heat treated to inactivate SIV and the set included a mixture of serum and plasma. Therefore, we started by optimizing the methodology and evaluating the effects of sample processing on antibody profiles. Profiling of macaque anti-glycan antibodies was done at a dilution of 1∶100 based on optimization studies. Typically, we profile human serum at a dilution of 1∶50, which provides high signal intensity without significant background. For macaque samples, a dilution of 1∶100 was required to reduce the average background to <1% of the scanner's dynamic range. As for sample processing, there were no significant differences in anti-glycan IgG or IgM antibodies measured before or after heat treatment (Figure S1 in File S1) and in plasma or serum (Figures S2–S3 in File S1).

**Figure 2:**
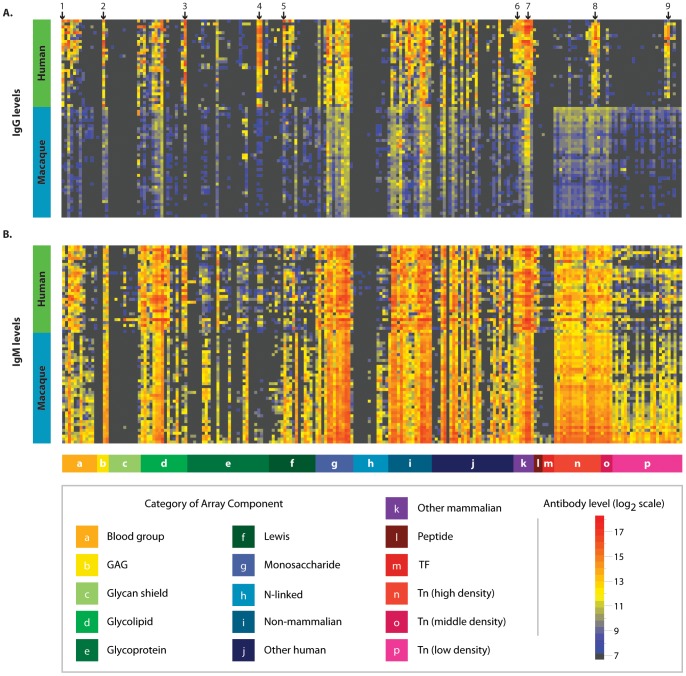
Comparison of anti-glycan antibody profiles in macaques and humans. Heat map showing glycan binding of circulating IgG (A) and IgM (B) in pre-vaccinated macaques (n = 38) and healthy humans (n = 30). Columns correspond to individual glycans organized into glycan families (see legend). Rows represent individual humans and macaques, which have been sorted by hierarchical clustering. Macaques and humans have highly similar repertoires of anti-glycan antibodies. Highlighted glycans are (1) 2′FucLac, (2) Hya8, (3) P1, (4) Ovalbumin, (5) LeA, (6) alpha-gal, (7) Forssman di, (8) Ac-Tn(Thr)-G-21, and (9) Ac-Tn(Thr)-Tn(Thr)-Tn(Thr)-G. Normalized data are plotted on a log 2 scale with a floor value of 7.2 (colored black).

Profiles of circulating anti-glycan antibodies of 38 macaques measured prior to vaccination were compared to the antibody profiles of 30 healthy humans. Although there were certain differences, macaques and humans shared similar overall profiles of anti-glycan antibodies (Figure 2). For instance, high levels of antibodies were found in both species for rhamnose-α/-β, glucose-β, cellotriose, a disaccharide substructure of blood group A, Forssman antigen, and alpha-gal. Although overall profiles were similar, some differences were noted. One of the largest differences between macaques and humans occurred for ovalbumin, presumably because humans are repeatedly immunized with egg derived vaccines. Differences were observed also for fucosylated lactose (2′FucLac), hyaluronic acid (Hya8), P1, Lewis A (LeA), and two glycopeptides [Ac-Tn(Thr)-G-21 and Ac-Tn(Thr)-Tn(Thr)-Tn(Thr)-G.] (Significant differences between humans and macaques are marked with arrows in Figure 2.) Overall, the similarities between the anti-glycan antibodies of macaques and humans support the use of macaques as a relevant model for studying anti-glycan antibodies.

### Widespread post-vaccination changes in anti-glycan IgM

We evaluated humoral responses to the vaccine by analyzing changes in anti-glycan IgG and IgM antibodies occurring after macaques were primed with various formulations of the Ad5hr –SIV vaccines followed by boosting with envelope immunogens (Table 1). Profiles of anti-glycan antibodies immediately prior to vaccination were compared to profiles at two weeks after completing the vaccine regimen but before SIV challenge. Since measurements of circulating anti-glycan antibodies in plasma and serum showed no significant difference (see Figures S2 and S3 in File S1), both serum and plasma samples were analyzed in order to increase the number of samples available to measure circulating antibody levels.

**Figure 3:**
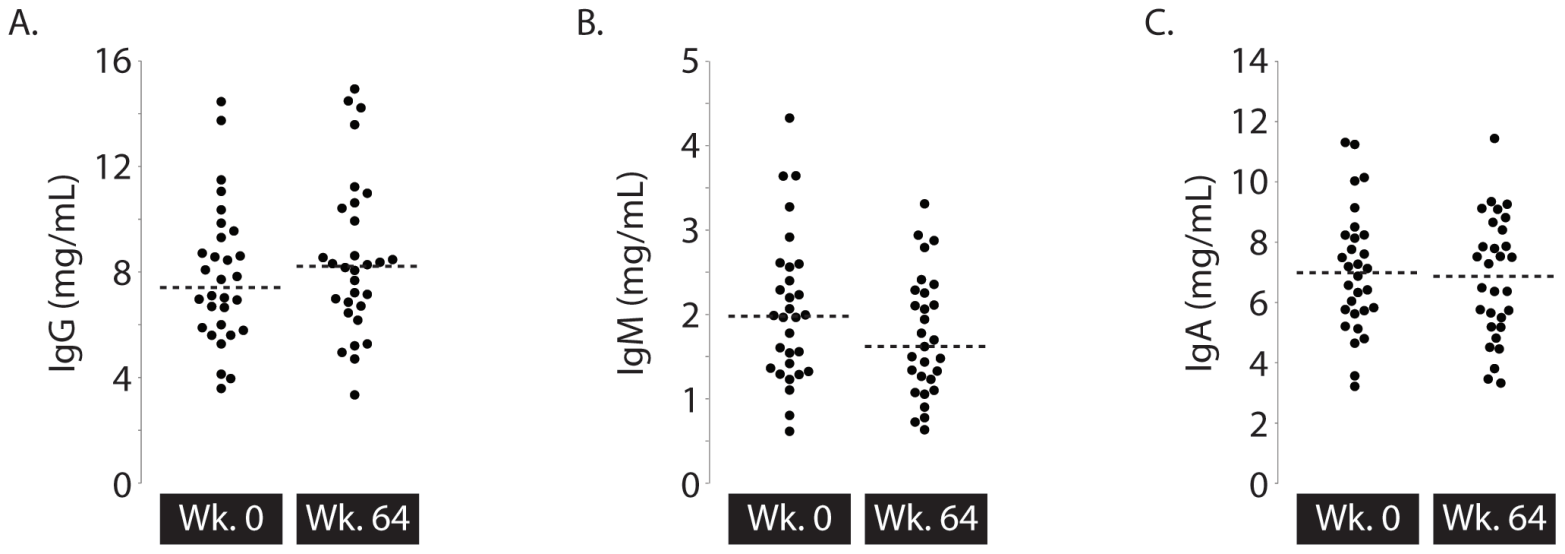
Changes in overall immunoglobulin levels. Overall levels of immunoglobulin (A  =  IgG, B  =  IgM, C  =  IgA) were measured in macaques (n = 30) at two time points – week 0 (prior to-vaccination and SIV challenge) and week 64 (after vaccination and SIV challenge). Dashed lines indicate the median values for each time point. P-values for comparison of the two time points were calculated with the Mann-Whitney test. Additionally, histograms show the fold changes in overall immunoglobulin (D  =  IgG, E  =  IgM, F  =  IgA) occurring after vaccination and SIV challenge.

A key factor in our analysis involved defining significant changes. We limited our analysis to post-vaccination changes of 4-fold and larger (i.e., increases of ≥300% and decreases ≥75%). This threshold was applied in order to focus on large changes in antibodies for specific glycans that are beyond normal physiologic variation or changes in total antibody levels. In a previous study of healthy humans [33], spontaneous changes of ≥2.6 fold occurred only rarely over a three month period, and this is the threshold that we typically use for human studies. However, this threshold may not be applicable to macaques that were vaccinated and challenged with SIV. To better define an appropriate threshold, we measured variations in total IgM, IgG, and IgA levels in each animal (Figure 3). Despite these individual changes, the ranges of immunoglobulin levels across all animals were similar before and after vaccination and challenge with SIV (Figure 3 A–C). There was no generalized change for the group, which suggests that changes for individual macaques occurred within a physiologic range. To analyze changes in immunoglobulin levels within individual animals, we calculated fold changes occurring from week 0 to week 64 (Figure 3D–F). Up to 4-fold changes were observed, but only one change of larger than 4-fold was detected (Figure 3E). Animal 9 had a 4.2 fold decrease in overall IgM levels. Since we were most interested in changes in specific anti-glycan antibodies that exceeded changes in overall immunoglobulin levels, we selected a threshold of 4-fold to avoid detecting changes due to general effects on immunoglobulin levels.

**Figure 4:**
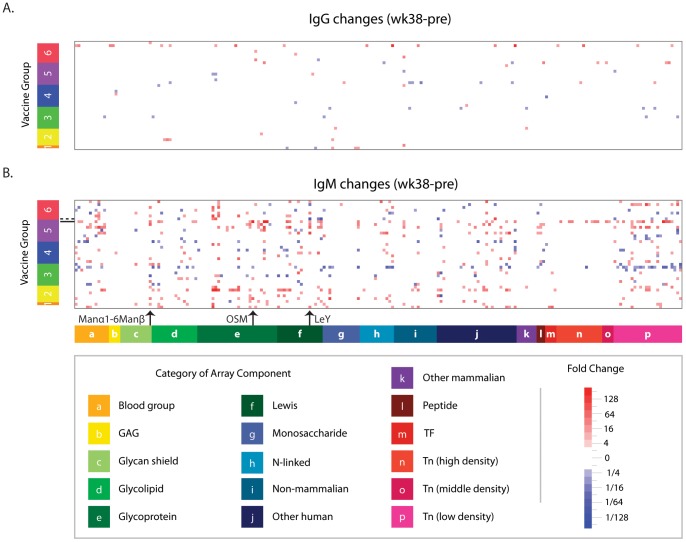
Post-vaccination changes in anti-glycan antibodies. Heat map showing changes in IgG (A) and IgM (B) circulating anti-glycan antibodies that occurred after vaccination (wk 38–wk 0). Rows correspond to individual macaques (n = 38) sorted by vaccine groups described in Table 1. Columns correspond to glycans grouped according to glycan family. Red indicates increases, and decreases are shown in blue. Non-significant changes (<4×) are white. As indicated by the arrows, Manα1-6Manβ had the most number of antibody changes, OSM had the largest increase in antibody levels, and LeY had the largest decrease. The dashed horizontal line marks the macaque with the large decline in anti-LeY IgM levels, and the solid line indicates the macaque with the largest OSM increase.

We began our analysis by generating a heat map (Figure 4) to provide an overview of changes in antibody levels across families of glycans. For many individual glycans, numerous 4-fold and larger changes were observed in anti-glycan antibodies after macaques received any of the vaccine regimens or control (unmodified adenovirus vector + MPL-SE adjuvant). IgM changes (392 increases : 164 decreases) outnumbered IgG changes (48 increases : 31 decreases). IgM changes were broadly dispersed over several classes of glycans. However, there was no predominant change that occurred in the majority of the animals. For each glycan, changes of ≥4 fold were seen for a small number of animals (mean number of animals showing change in antibody levels to any single glycan  = 2.6, median = 1, and mode = 0). The glycan with the largest frequency of change was Manα1-6Manα, for which 11 animals had changes (29%) of ≥4-fold (Figure 4B). The largest increase (375-fold) was seen for ovine submaxillary mucin (OSM, Figure 4B), which is a glycoprotein with numerous O-linked glycans. The largest decrease (32-fold) occurred for Lewis Y (LeY, Figure 4B).

Although there were numerous changes in anti-glycan levels, there were no significant differences between animals receiving control vector and animals receiving an HIV vaccine (Figure 4). Moreover, there were no significant differences across groups of vaccinated animals. Of special note, there were no statistically significant differences for group 5, which received the non-glycosylated peptomer in PBS rather than glycosylated gp120 in MPL-SE. Based on these results, the observed changes in anti-glycan antibodies are likely due to the Ad5hr viral vector rather than responses induced by the SIV transgenes, protein boosts, or adjuvant. It should be noted, however, that due to the large number of possible comparisons between the many vaccine groups and the number of available samples, small but statistically significant differences between vaccine groups would be difficult to detect.

### Few post-vaccination responses to the glycan shield

Humoral responses to the glycan shield have been proposed to be one aspect of a protective response to vaccination. Thus, we specifically analyzed humoral responses to 11 glycans (Figure S4 in File S1) known to be components of the glycan shield. These glycans included several complex high-mannose glycans similar to the carbohydrate antigen recognized by the broadly cross-neutralizing antibody 2G12 [46]. Although there were numerous changes in anti-glycan antibodies across many classes of self and non-self glycans, there was no indication of a prominent humoral response to any component of the glycan shield (Table S2 in File S1).

### Early humoral responses to SIV challenge (wk 38 vs wk 46)

Six weeks after completing the vaccination regimen, macaques were challenged on week 42 with 10 MID_50_ of SIV_mac251_. Anti-glycan antibodies were profiled at 4 and 22 weeks after SIV challenge (overall weeks 46 and 64). In addition to our primary aim of characterizing the frequency and diversity of anti-glycan antibody responses occurring after SIV infection, we looked for correlations between post-challenge, anti-glycan humoral responses and ability to control SIV infection, as indicated by viral loads and CD4^+^ T-cell counts. In particular, we were interested in humoral responses that could discriminate elite controllers (n = 5 of 38) that suppressed SIV infection as evident from low or undetectable SIV levels and normal CD4^+^ T-cell counts.

**Figure 5:**
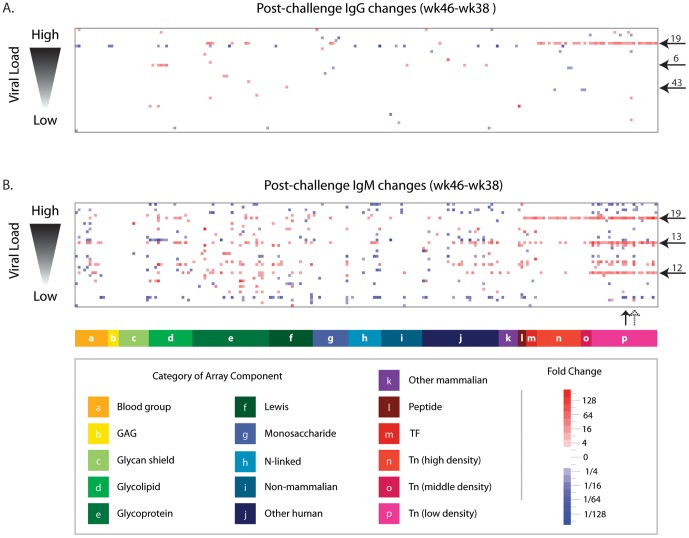
Early changes in anti-glycan antibody levels after SIV infection. Heat map showing changes in IgG (A) and IgM (B) circulating anti-glycan antibodies that occurred after vaccinated macaques were challenged with SIV (wk 46–wk 38). Rows correspond to individual macaques (n = 38) sorted according to their ability to control SIV infection, as indicated by viral load measured at week 64 Columns are glycans grouped by category. Non-significant changes (<4×) have been colored white. Five macaques (animals 6, 12, 13, 19, and 43) with the most consistent Tn responses are marked with horizontal arrows. Vertical arrows indicate two Tn glycopeptides (solid arrow is Ac-S-Tn(Thr)-S-G-04 and dashed arrow is Ac-S-Tn(Thr)-V-G-04), which had changes occurring in the largest number of macaques (32%).

In the acute phase of infection [four weeks after challenge (wk 46) relative to post-vaccination (wk 38)], substantial changes in anti-glycan antibodies were evident (Figure 5). Macaques showed an approximately balanced number of IgM changes (335 increases : 222 decreases). Fewer IgG changes occurred (74 increases : 39 decreases). Changes tended to vary substantially from one animal to another, with the most common change observed across animals being IgM responses to two Tn glycopeptides (Ac-S-Tn(Thr)-S-G-04 and Ac-S-Tn(Thr)-V-G-04 in 32% of animals). While numerous anti-glycan antibody changes were observed in this phase of infection, none showed a statistically significant correlation with outcomes (viral load and CD4^+^ levels).

Interestingly, several animals developed broad, prominent responses to the Tn antigen, which consists of a single GalNAc monosaccharide alpha linked to a serine or threonine of a polypeptide chain. Although the Tn antigen is best known as a tumor-associated carbohydrate antigen, some studies have reported that monoclonal antibodies to the Tn antigen neutralize HIV by preventing fusion between HIV-infected and uninfected cells [47], [48]. The glycan microarray used in this study contained many Tn glycopeptides with various peptide sequences and densities of Tn. We found changes (both increases and decreases) in antibody binding to many Tn glycopeptides, especially when presented at low density. By four weeks after challenge (overall week 46), one viremic macaque (animal 19) mounted ≥4× IgG increases to 35 of the 39 Tn glycopeptides on the array. The largest fold increase (15×) was for Ac-A-Tn(Thr)-S-G – 08. For 18 Tn glycopeptides, the post-challenge anti-Tn IgG levels for animal 19 were higher than the normal range measured in non-vaccinated macaques, which suggests that the Tn responses were not due only to variation within the normal range. Animal 19, along with two other viremic macaques, mounted IgM increases to nearly all low density Tn glycopeptides.

Interestingly, Tn responses occurred more frequently than responses to the glycan shield. SIV challenge induced a number of anti-glycan antibody changes, but humoral responses to the glycan shield were rare four weeks post-challenge (Table S2 in File S1). Seven macaques showed changes in IgM antibody levels for one disaccharide fragment (Manα1-6Manα) found in some high mannose glycans. Immune responses to only one other glycan shield antigen (IgM response to Man6-II in 2 macaques) were seen. Antibody levels to the other 9 mannose containing antigens were not significantly changed in any of the 38 animals.

### Later humoral responses to SIV challenge (wk 38 vs. wk 64)

**Figure 6:**
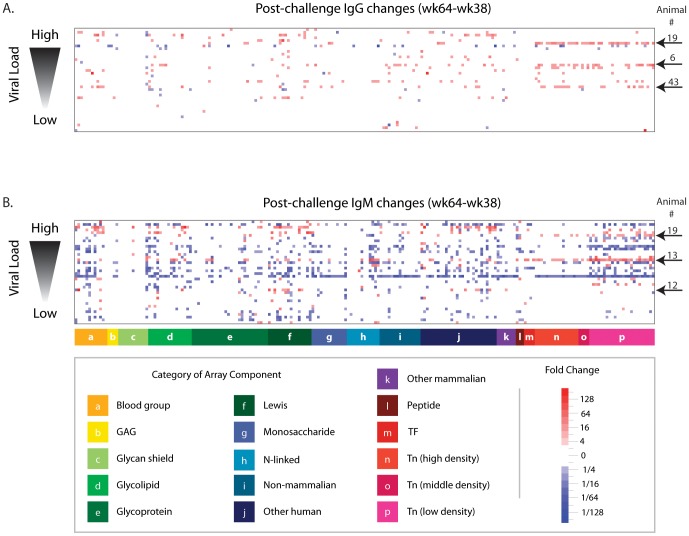
Later changes in anti-glycan antibody levels after SIV infection. Post-infection changes in anti-glycan antibody levels were repeated for overall week 64 (22 weeks after challenge). Heat map showing changes in circulating IgG (A) and IgM (B) anti-glycan antibodies that occurred after vaccinated macaques were challenged with SIV (wk 64–wk 38). Rows correspond to individual macaques (n = 38) sorted according to their ability to control SIV infection, as indicated by viral load measured at week 64. Columns are glycans grouped by category. White indicates non-significant changes (<4×). Five macaques (animals 6, 12, 13, 19, and 43) with the most consistent Tn responses are marked with arrows.

**Figure 7:**
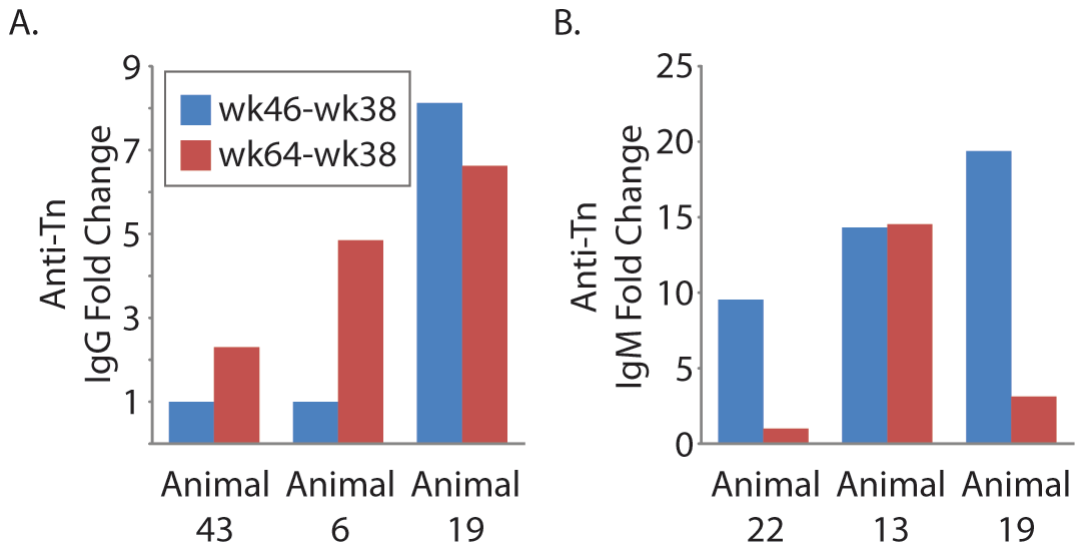
Post-vaccination Tn responses. Five of the 38 macaques showed increases in anti-Tn IgG (A) and/or IgM (B). For these 5 animals, blue bars indicate the average fold change for 23 low density Tn glycoproteins occurring within 8 weeks of SIV infection (overall week 46–week 38). Red bars show the fold change after 26 weeks of infection (overall week 64–week 38). The animals are ordered in increasing levels of viremia.

Anti-glycan antibody levels were profiled during the chronic phase of infection, 22 weeks after SIV challenge (overall week 64). Compared to the earlier post-challenge time point, more changes were observed in IgG and IgM antibody levels at the later time point (Figure 6). Anti-Tn antibodies again showed some of the largest and most frequent changes (Figure 7A). The anti-Tn IgG response seen in one viremic animal (#19) at week 46 persisted to week 64. Also, another two viremic animals showed anti-Tn IgG increases between weeks 46 and 64. Once again, levels of Tn antibodies measured after SIV challenge exceeded the normal range measured in non-vaccinated macaques, which suggests that Tn changes were larger than normal physiologic variation or technical error. In animal 19, antibody levels changed more than four-fold for 34 of the 39 Tn glycopeptides present on the array. For 24 of the changes, the post-challenge antibody levels exceeded the normal range measured at week 0. Sixteen of animal 6′s 23 changes exceeded the normal range. One of 12 significant changes exceeded the normal range for animal 43. As further indication that the Tn responses were not artifact due to physiologic variation or technical error, no macaques showed consistent declines in Tn-antigen. Whereas physiologic variation and technical error would be equally likely to cause increases and decreases, we observed only significant increases in anti-Tn antibodies.

As the period of SIV infection increased and some animals progressed to chronic viremia (week 64), there was a generalized decrease in anti-glycan IgM antibodies [Figure 6B; decreases (n = 796) outnumbered increases (n = 284)]. However, similar decreases in total IgM levels were not observed, indicating a unique effect in the subset of serum antibodies that recognize glycans. Once again, decreases were noted across diverse classes of glycans, such as self, bacterial, and plant glycans. Levels of anti-Tn IgM were affected, and the Tn response at week 46 declined for two animals (Figure 7B). Interestingly, the substantial decrease in antibodies for IgM as SIV infection progressed was not seen for IgG, for which increases (n = 259) outnumbered decreases (n = 62).

Additionally, we looked for correlations between antibody responses and outcomes (viral load and CD4^+^ levels). Although the correlations did not reach statistical significance in this small study, anti-glycan antibody levels consistently tended to be more dynamic in viremic animals relative to elite controllers. Viremic animals tended to have the most prominent decreases in anti-glycan IgM antibodies after prolonged infection, and the large decreases in IgM antibody levels were not seen in the elite controllers. Antibody levels tended to be more dynamic in viremic animals (n = 33) than in elite controllers (n = 5). Across the entire array (n = 220 features), viremic animals on average showed 8.3 IgM increases compared to an average of 2.2 IgM increases for elite controllers (p = 0.09 by Mann-Whitney non-parametric test). Similarly, each viremic animal averaged 7.6 IgG increases over the entire array, whereas elite controllers averaged 1.4 IgG increases (p = 0.53). The average number of IgM decreases across the entire array was 21.9 for viremic animals and 14.6 for elite controllers (p = 0.84). Finally, viremic animals showed an average number of 1.8 IgG decreases relative to 0.6 IgG decreases for elite controllers (p = 0.97). Low levels of CD4^+^ T cells also tended to be associated with larger and more frequent decreases in IgM antibodies (Figure S5 in File S1).

## Discussion

Development and optimization of an effective HIV vaccine requires a detailed understanding of the immune response and the elements that contribute to efficacy. Although relatively understudied, anti-glycan humoral responses are an especially important aspect of HIV immunity, as illustrated by neutralizing antibodies that recognize the glycan shield of HIV. Since anti-glycan antibodies are a distinct subpopulation of antibodies that do not adhere to the classical paradigm of the adaptive immune response, it is important to determine how HIV vaccines and challenge affect anti-glycan antibodies levels, if at all. Here, we report the first comprehensive profiling of circulating anti-glycan antibody responses to SIV vaccination and SIV challenge in a non-human primate model of HIV infection. This work adds to knowledge about humoral responses to SIV vaccination and infection by focusing on responses to glycans, which are predominantly T-cell independent antigens [49], [50].

Vaccination and SIV challenge induced significant changes in anti-glycan antibody repertoires. Changes induced by vaccination were likely general effects resulting from the viral vector, as similar responses were observed in vaccinated and control animals regardless of gp120 or peptomer boost and whether these Env immunogens were formulated in MPL-SE adjuvant or PBS. SIV challenge induced several unexpected changes. In particular, responses to a tumor-associated carbohydrate antigen, the Tn antigen (GalNAc alpha-linked to serine or threonine), were observed in a number of animals. Responses included both IgM and IgG changes. Although broad Tn responses were observed in a relatively small number of animals after vaccination, these responses do not appear to be a result of the viral vectors or the adjuvants or simply due to normal variation over time. Therefore, additional studies on responses to the Tn antigen and warranted.

Anti-Tn antibodies may be induced by expression of Tn on SIV. Although the Tn antigen is best known as a tumor associated carbohydrate antigen, it has also been reported as a neutralizing epitope on HIV [51]. Tn is a precursor for many O-glycans, which have been found on HIV [52], [53], [54] and SIV [55]. In healthy cells, the glycan chain is typically extended, thereby masking the Tn antigen on glycoproteins before their arrival on the cell surface. When glycosylation is perturbed, premature termination of glycan chains can expose the Tn antigen. Early escape of the viral particle from the normal glycosylation pathways within the Golgi apparatus may cause premature termination of *O*-glycans, thereby revealing the Tn antigen on SIV or HIV. Such a mechanism is consistent with HIV envelope modifications that occur as patients progress from asymptomatic chronic infection to end-stage AIDS. These changes include decreases in potential *N*-linked glycosylation sites (PNGS) and an increase in overall positive charge [56]. However, little is known regarding the glycan motifs that occupy the individual PNGS. A comparison of HIV transmitted-founder isolates obtained during acute infection and isolates obtained during chronic infection has shown distinct differences including a greater percentage of variably utilized or unutilized sites, and a greater proportion of sites with high mannose glycans as opposed to complex or highly processed glycans [57], [58], [59]. These isolates were expressed in 293 cells, however, and protein glycosylation varies with the host cell or cell line used for expression [60], [61]. Glycan motifs that occur *in vivo* with disease progression are not known, and further studies are needed to confirm expression of Tn on SIV.

Interestingly, only minimal responses to the glycan shield were observed following vaccination or infection. Responses to the glycan shield, which typically develop after prolonged viral infection, may not have been observed due to the short time frame of this study. Since broadly neutralizing antibodies typically arise two to three years after HIV infection in humans, the time frame of this study may have been too short to observe development of antibodies to the glycan shield. Broadly neutralizing antibodies such as VRC01 undergo multiple rounds of affinity maturation and are highly mutated compared to their germ line predecessors [62], indicating a potential need for constant antigen exposure over time that was not achieved here by 2 weeks post-immunization or by 22 weeks post-challenge.

Anti-Tn responses occurred despite a generalized, post-infection decline in anti-glycan IgM antibodies in some macaques. Although anti-glycan antibodies are generally recognized as natural antibodies that are characteristically invariant to stimuli [63], SIV induced widespread changes in antibodies that bind to diverse glycans. Moreover, generalized decreases in anti-glycan antibodies are somewhat unexpected because HIV is associated with hypergammaglobulinemia [64], [65], [66], which is thought to involve depletion of memory B cells accompanied by plasmacytosis of naïve B cells [65]. IgM for self-antigens has been postulated to increase at the expense of non-self antibodies [66], which have a greater dependence on CD4^+^ helper T-cells. HIV is associated with increases in total IgG [65], whereas IgM levels remain stable or increase [67]. While total levels of IgM measured in this study fell into the range of immunoglobulin concentrations of healthy animals [68], the subset of IgM that recognizes glycans showed significant decreases in a number of animals. Interestingly, these decreases were observed predominantly in viremic animals and were not observed in elite controllers.

The generalized decrease in anti-glycan IgM seen for several viremic macaques may result from B-cell dysregulation, which is a hallmark of SIV infection [69]. We have reported the continued loss of activated memory B-cells from PBMC and bone marrow of SIV-infected rhesus macaques even during ART [70]. HIV also produces B-cell dysfunction. In human PBMC, a subpopulation of memory B-cells, known as B1 cells, produce natural antibodies, which are broadly reactive and often autoreactive [71]. In HIV infection, antibody polyreactivity increases [72], and anti-cardiolipin antibodies have been described in 49%–58% of HIV infected patients [73]. In fact, the levels of autoreactive antibodies have been associated with membrane proximal external region (MPER) binding antibodies such as 4E10, isolated from an HIV infected patient, which binds to cardiolipin [74]. It is possible that the decreases in anti-glycan IgM antibodies reflect class-switching of IgM natural antibodies to either IgA or IgG. Given the small number of elite controllers (n = 5), additional studies are warranted to further investigate this hypothesis and more fully evaluate the association of decreases in IgM levels with viremia and low levels of CD4^+^ T cells.

The antibody responses to the Tn antigen and generalized decline in anti-glycan IgM antibodies observed in this study are intriguing, but additional studies will be needed to extend these results to HIV infection. Although we show that humans and macaques have similar overall anti-glycan antibody repertoires, these species might differ in the production, regulation, or function of these antibodies. Since SIV progresses more rapidly than HIV, this study also assessed antibody levels over a shorter time frame than HIV typically requires to produce immunodeficiency. Therefore, it will be useful to evaluate anti-glycan responses in humans and assess changes over a longer period of time.

Taken together, this hypothesis generating study illustrates the potential of using glycan microarrays to study anti-glycan antibodies in SIV and HIV. Anti-glycan antibody levels do change in response to both SIV vaccination and infection, and glycan microarrays can be used to measure these changes in a relevant non-human primate model of HIV. Importantly, these new insights into an underexplored aspect of SIV immunity highlight the potential for glycan microarrays to contribute to vaccine development. Given our results, additional studies on anti-glycan antibody responses induced by vaccination and HIV infection in humans are warranted.

## Supporting Information

File S1 **Supporting Tables and Figures.**
(PDF)

File S2 **Microarray Data and Array Description.**
(XLSX)
