# Correction: High-Throughput Profiling of Anti-Glycan Humoral Responses to SIV Vaccination and Challenge

**DOI:** 10.1371/annotation/fd9f4a65-3f27-46cd-8ed0-6c9b8ba7f83b

**Published:** 2013-12-03

**Authors:** Christopher T. Campbell, Sean R. Llewellyn, Thorsten Demberg, Ian L. Morgan, Marjorie Robert-Guroff, Jeffrey C. Gildersleeve

The name of the third author was spelled incorrectly. The correct name is: Thorsten Demberg. The correct Citation is: Campbell CT, Llewellyn SR, Demberg T, Morgan IL, Robert-Guroff M, et al. (2013) High-Throughput Profiling of Anti-Glycan Humoral Responses to SIV Vaccination and Challenge. PLoS ONE 8(9): e75302. doi:10.1371/journal.pone.0075302. 

