# Supplementary material for: High-Throughput Profiling of Anti-Glycan Humoral Responses to SIV Vaccination and Challenge
Source: PLoS One. 2013 Sep 23;8(9):e75302. doi: 10.1371/journal.pone.0075302 (PMC3781036; doi:10.1371/journal.pone.0075302)
Supplement: File S1 — Supporting Tables and Figures. (PDF) [file pone.0075302.s001.pdf]

Supplementary Materials

for

High-throughput profiling of anti-glycan humoral responses to SIV vaccination and challenge

Authors: Christopher T. Campbell[1], Sean R. Llewellyn[1], Thorsten Damberg[2], Ian L. Morgan[2], Marjorie Robert-Guroff[2]*, Jeffrey C. Gildersleeve[1]*

Author Affiliations:

1. Chemical Biology Laboratory, National Cancer Institute, National Institutes of Health, Frederick, MD 21702
2. Vaccine Branch, National Cancer Institute, National Institutes of Health, Bethesda, MD 20892

***Corresponding Authors:**

Jeffrey C. Gildersleeve
376 Boyles Street
Room 208
Frederick, MD 21702
301-846-5699
gildersj@mail.nih.gov

Marjorie Robert-Guroff
41 Medlars Drive MSC5065
Building 41, Room D804
Bethesda, MD 20892-5065
301-496-2114
guroffm@mail.nih.gov

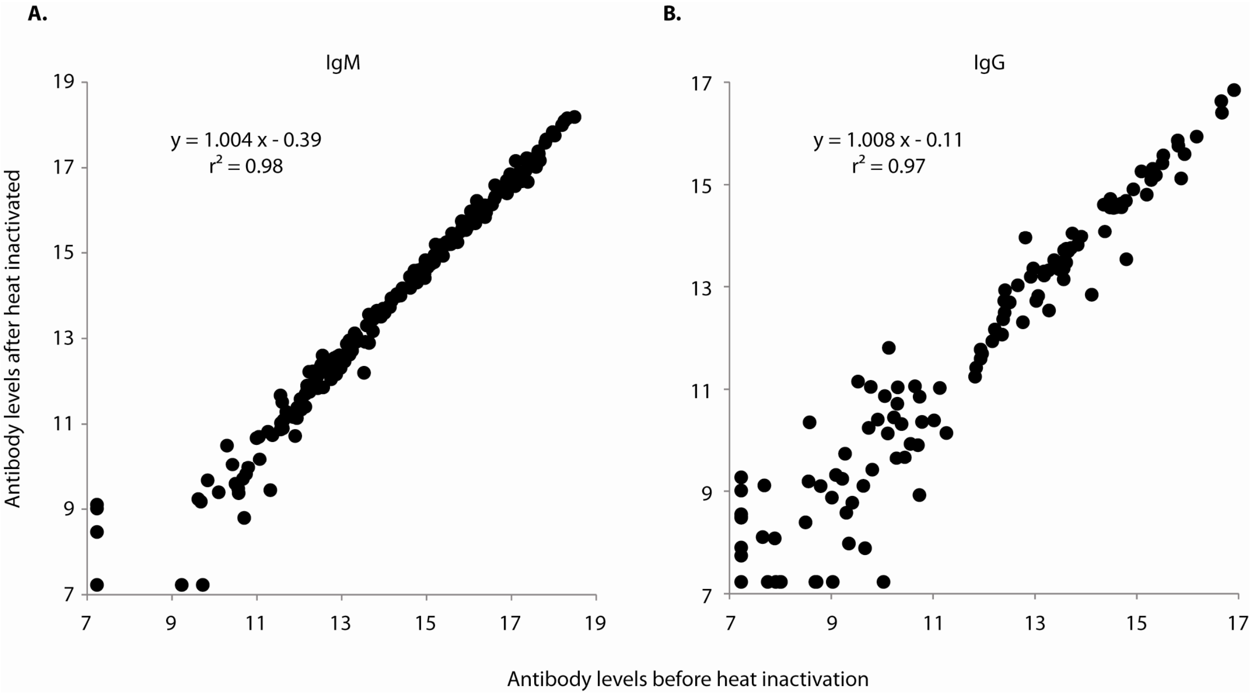

Supplemental Fig. S1. Effect of heat treatment on anti-glycan antibody levels. Scatterplots comparing levels of anti-glycan (A) IgM and (B) IgG antibodies before and after heat inactivation of SIV (56^{0}C for 30 minutes).

Secondary Antibody	Supplier	working concentration
**TRITC Goat anti-monkey IgM**	Brookwood Biomedical (Catalog # SAB1157)	2 µg/mL
**TRITC Goat anti-monkey IgG**	Brookwood Biomedical (Catalog # SAB1307)	2 µg/mL

Supplemental Table S1. Secondary antibodies for detection of IgM and IgG

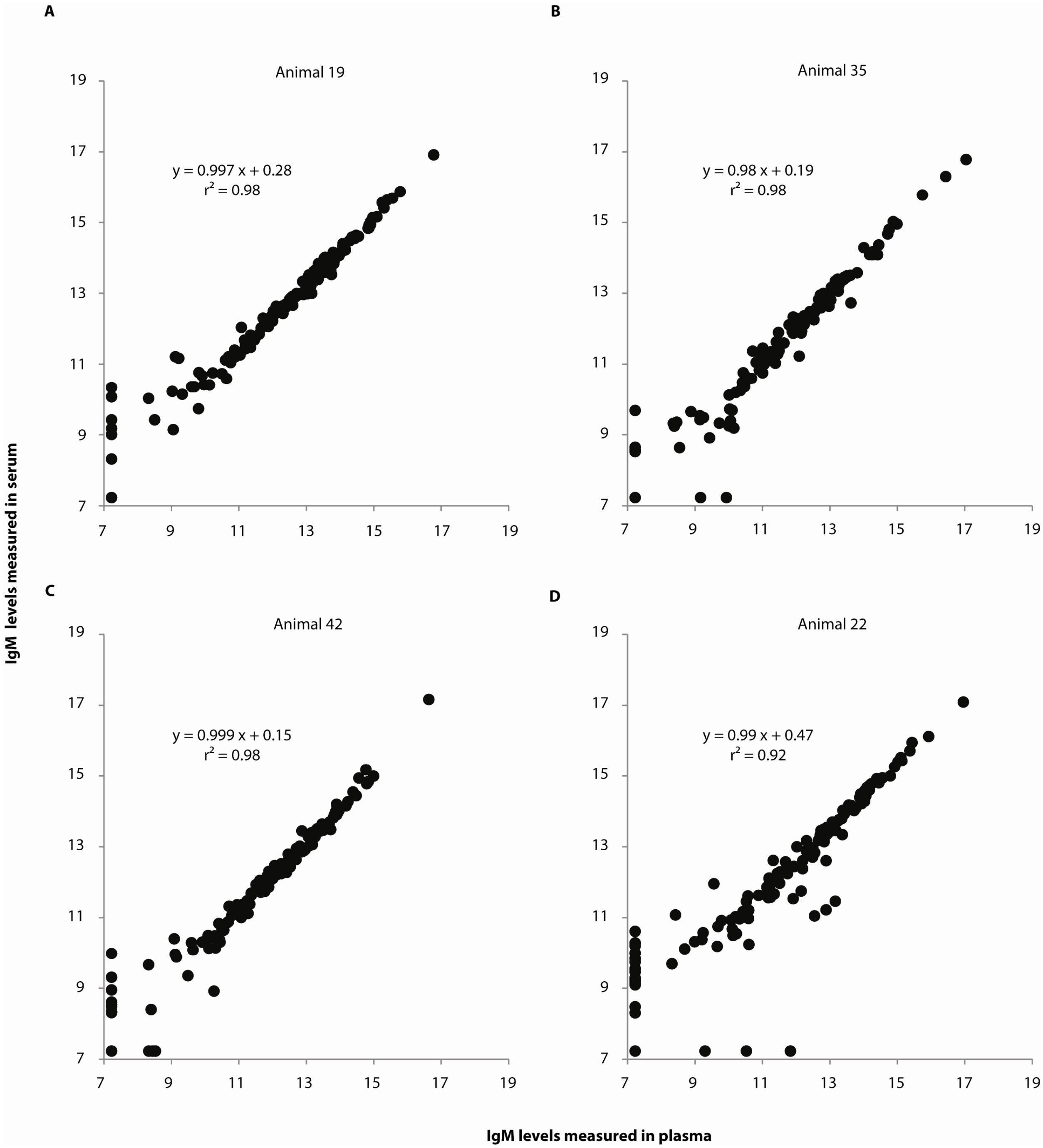

Supplemental Figure S2. Levels of circulating anti-glycan IgM measured in plasma and serum. Serum and plasma samples were obtained simultaneously from 4 macaques (A-D). For each animal, scatterplots compare the levels of IgM bound to each array component measured in plasma (x-axis) with the corresponding levels of IgM antibody measured in serum (y-axis).

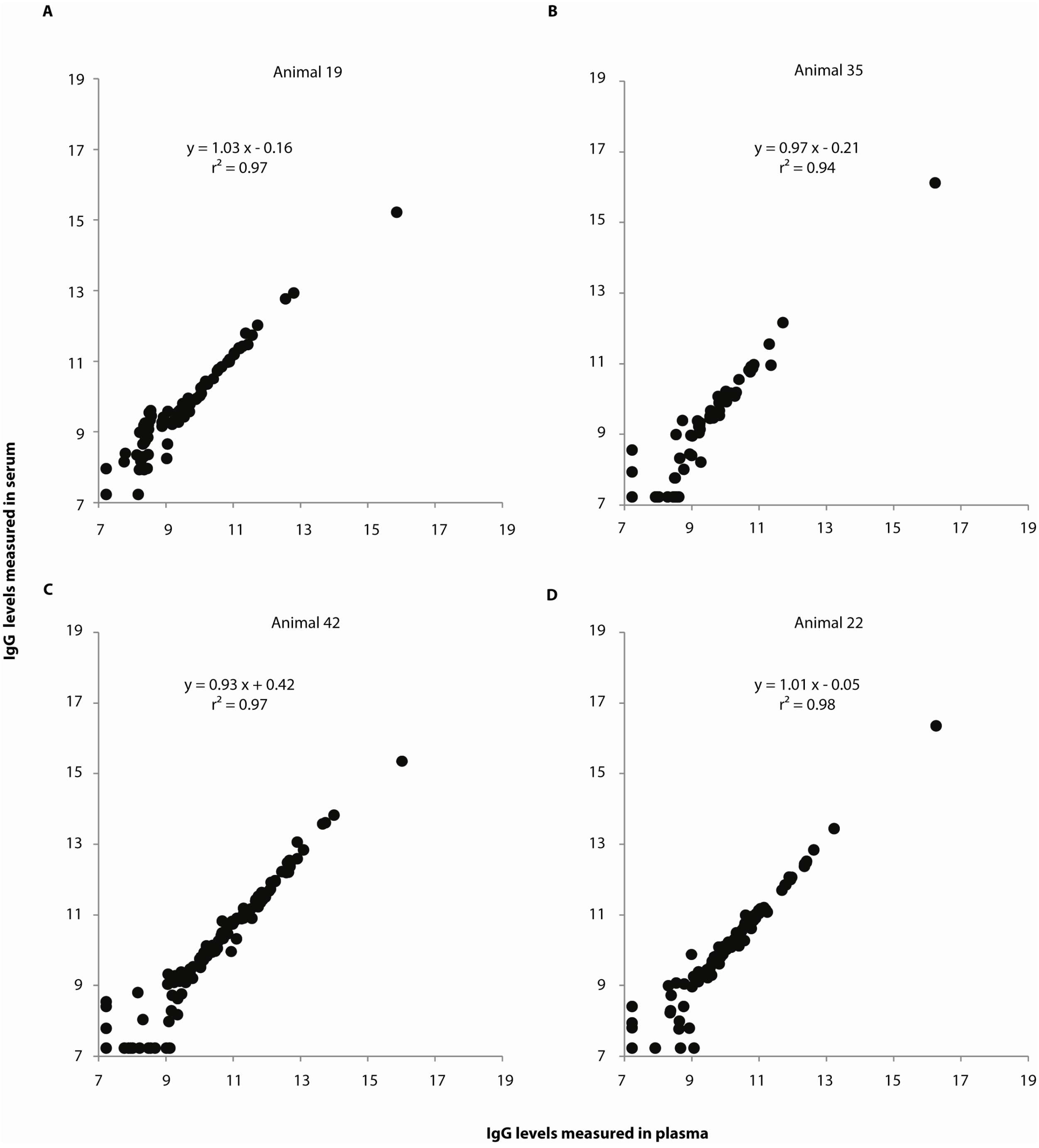

Supplemental Figure S3. Levels of circulating anti-glycan IgG measured in plasma and serum. Serum and plasma samples were obtained simultaneously from 4 macaques (A-D). For each animal, scatterplots compare the levels of IgG bound to each array component measured in plasma (x-axis) with the corresponding levels of IgG antibody measured in serum (y-axis).

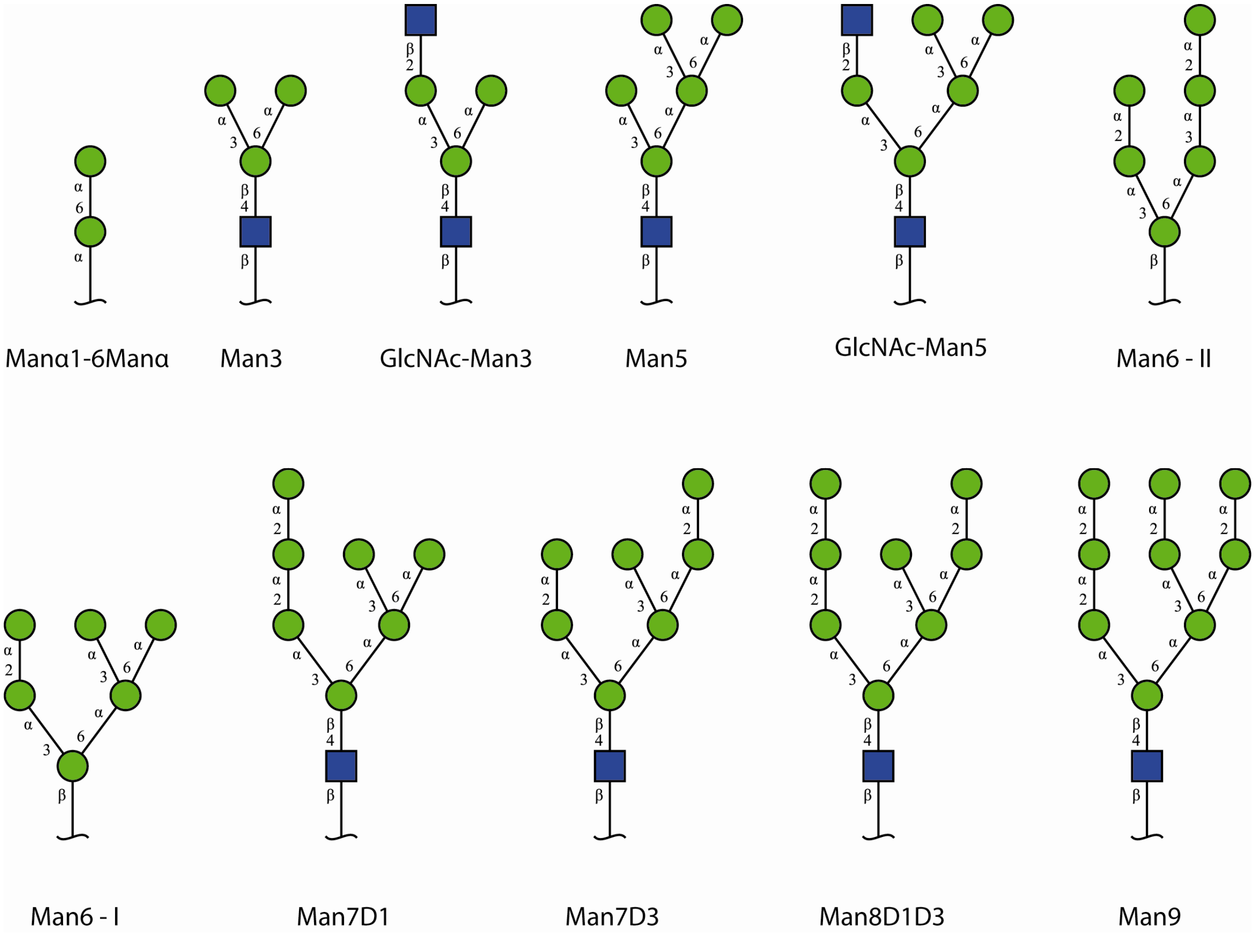

Supplemental Figure S4. Array components related to the glycan shield of HIV. The glycan microarray contained 11 components reported to be fragments of the glycan shield.

Glycans	Isotype	Post-Vaccination		4 Wks Post-Challenge		22 Wks Post-Challenge	
		Increase	Decrease	Increase	Decrease	Increase	Decrease
Manα1-6Manα	IgM	10	1	2	5	2	14
	IgG	1	0	0	0	1	5
Man3	IgM	0	0	0	0	0	0
	IgG	0	0	0	0	0	0
GlcNAc-Man3	IgM	0	0	0	0	0	0
	IgG	0	0	0	0	0	0
Man5	IgM	0	0	0	0	0	0
	IgG	0	0	0	0	0	0
GlcNAc-Man5	IgM	0	0	0	0	0	0
	IgG	0	0	0	0	0	0
Man6 - I	IgM	0	0	0	0	0	0
	IgG	0	0	0	0	0	0
Man6 - II	IgM	3	1	1	1	3	1
	IgG	0	0	0	0	0	0
Man7D1	IgM	0	0	0	0	0	0
	IgG	0	0	0	0	0	0
Man7D3	IgM	0	0	0	0	0	0
	IgG	0	0	0	0	0	0
Man8D1D3	IgM	0	0	0	0	0	0
	IgG	0	0	0	0	0	0
Man9	IgM	0	0	0	0	0	0
	IgG	0	0	0	0	0	0

Supplementary Table S2. Changes in antibody levels for components of the glycan shield.

Changes in antibodies for components of the glycan shield rarely occurred in any of the 38

macaques after vaccination with Ad5hr-SIV (wk 0 vs. wk 38) or following challenge with SIV (wk 38 vs. wk.46 & wk. 38 vs. week 64). The number of macaques that showed four-fold changes (increases ≥300% or decreases ≤75%) in anti-glycan antibodies are summarized for each of the 11 array components shown in Fig. 5.

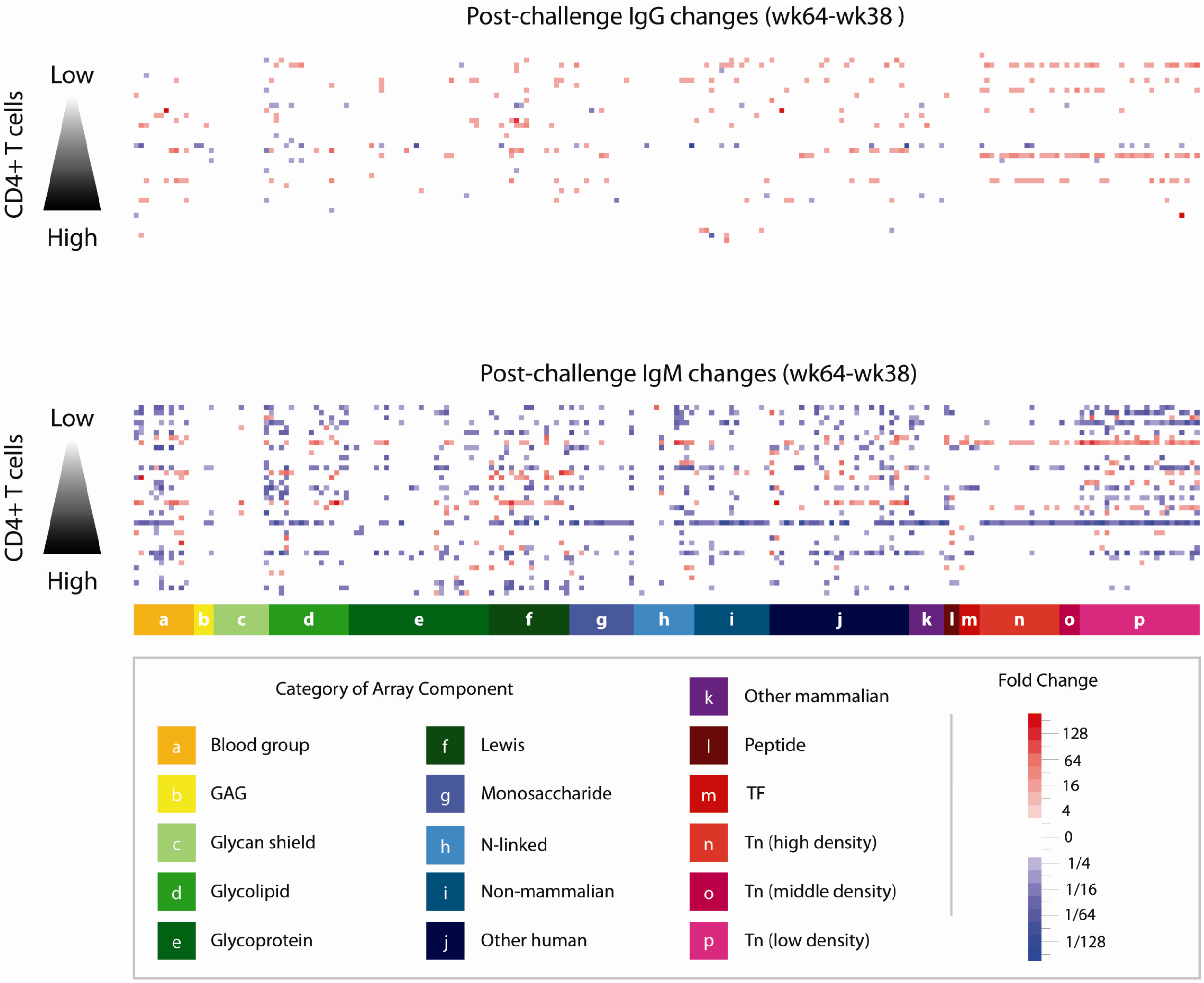

Supplemental Figure S5. Later changes in anti-glycan antibody levels after SIV infection. Post-infection changes in anti-glycan antibody levels were analyzed for overall week 64 (22 weeks after challenge). This heat map shows changes in circulating IgG (A) and IgM (B) anti-glycan antibodies that occurred after vaccinated macaques were challenged with SIV (wk 64 – wk 38). Rows correspond to individual macaques (n=38) sorted according to progression of SIV infection, as indicated by CD4+ T cell levels measured at week 64. Columns are glycans grouped by category. White indicates non-significant changes (<4x).
